# Non-operative management of barium peritonitis: a case report

**DOI:** 10.1093/jscr/rjaf633

**Published:** 2025-11-12

**Authors:** Muhammad Mohsin Khan, Abdullah Bin Faisal, Rabia Chang, Usama Iqbal

**Affiliations:** Medical College, Aga Khan University, Stadium Road, Karachi 34800, Sindh, Pakistan; Medical College, Aga Khan University, Stadium Road, Karachi 34800, Sindh, Pakistan; Department of Surgery, Aga Khan University, Stadium Road, Karachi 34800, Sindh, Pakistan; Department of Surgery, Aga Khan University, Stadium Road, Karachi 34800, Sindh, Pakistan

**Keywords:** barium peritonitis, non-operative management, ileostomy reversal, CT-guided drainage, contrast agent safety

## Abstract

Barium peritonitis is a rare but serious complication caused by leakage of barium sulfate into the peritoneal cavity during gastrointestinal contrast studies. It carries high mortality and often necessitates surgical intervention. A 33-year-old female developed chemical peritonitis following a barium loopogram performed during her ileostomy reversal workup. Imaging revealed pneumoperitoneum and intra-abdominal collections due to barium spillage. Despite radiological findings, she remained clinically stable with only localized symptoms. Conservative management was pursued with intravenous antibiotics, hydration, analgesia, and CT-guided drainage. Surgery was avoided due to her stable clinical status and normal inflammatory markers. She recovered completely without in-hospital complications and remained asymptomatic on follow-up. This case demonstrates that conservative management of barium peritonitis is a viable option in selected stable patients. It also highlights the need to reconsider the use of barium-based contrast agents in favor of safer alternatives, especially in high-risk individuals.

## Introduction

Barium peritonitis is characterized by severe inflammation of the peritoneum due to unwanted leakage of barium into the abdominal cavity. This occurs most commonly as a consequence of radiological studies that involve a barium contrast. Although generally inert within the human bowel, the accidental leakage of barium into the peritoneal cavity due to undiagnosed perforations can lead to very severe and at times fatal complications. Signs and symptoms of peritonitis such as abdominal guarding and rigidity, vomiting, lethargy/anorexia, hypotension, and tachycardia have been previously reported [1, 2]. Development of granuloma and barytoma are also known to occur [3].

Disease mortality is significant for this pathology ranging from 20% to 53%, although numbers have shown to be aggravated if patients develop concurrent bacterial infection of the peritoneum [4].

We present the case of a young female patient who developed barium peritonitis following a barium loopogram done as part of an ileostomy reversal procedure, who was successfully conservatively managed and underwent complete recovery.

## Case presentation

A 33-year-old woman with no known comorbidities presented to the emergency department with a 1-week history of abdominal pain, distension, vomiting, and fever. These symptoms developed following a barium loopogram performed at another hospital as a part of the preoperative workup for ileostomy reversal. Her medical history revealed bowel perforation secondary to typhoid fever 2 months prior, for which she underwent an exploratory laparotomy and diverting ileostomy at the previous facility where she was being treated. Operative findings at that time revealed a 1 × 1 cm ileal perforation located at the antimesenteric border of the ileum, ~2.5 feet proximal to the ileocecal junction.

At the time of her presentation, she was hemodynamically stable, conscious, alert, and awake. Abdominal examination on inspection revealed a right-sided healthy loop ileostomy, with stoma bag attached. Upon palpation, rigidity, guarding, and tenderness localized to the left lower quadrant were noted. Examination of the cardiovascular, respiratory, and central nervous systems was unremarkable.

**Figure 1:**
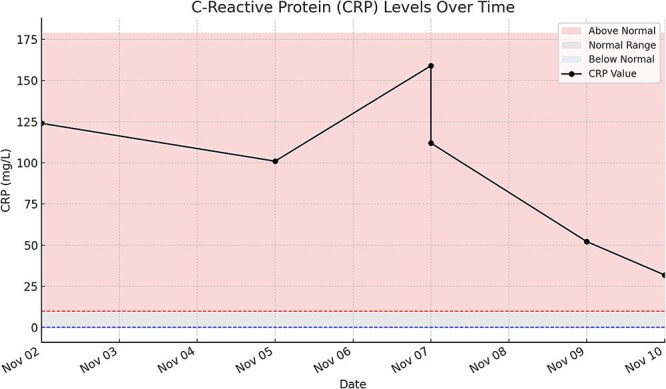
Line with markers—CRP values measured over time. Red shaded area—CRP levels above normal range (>10 mg/l). Gray shaded area—CRP levels within the normal reference range (1–10 mg/l). Blue shaded area—CRP levels below normal (<1 mg/l). Red dashed line—upper limit of normal CRP (10 mg/l). Blue dashed line—lower limit of normal CRP (1 mg/l).

Her C-reactive protein (CRP) was elevated at presentation (124 mg/l). Four days later, a drain was placed, after which her CRP peaked at 159 mg/l the following day, before declining to 112 mg/l later the same day. Two days prior to discharge, her CRP had decreased markedly to 31.9 mg/l, reflecting a significant reduction from admission (Fig. 1).

**Figure 2:**
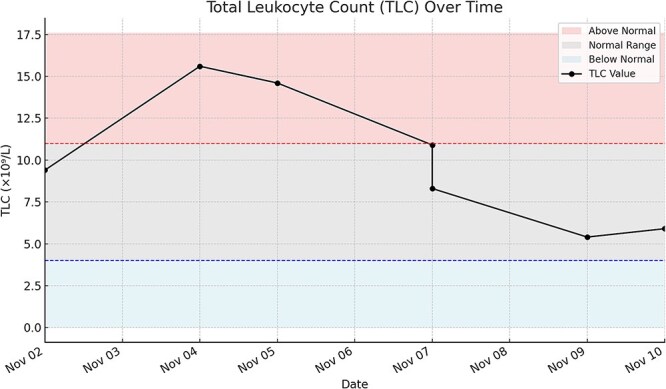
Line with markers—TLC values measured over time. Red shaded area—TLC levels above normal range (>11 × 10^9^/l). Gray shaded area—TLC levels within the normal reference range (4–11 × 10^9^/l). Blue shaded area—TLC levels below normal (<4 × 10^9^/l). Red dashed line—upper limit of normal TLC (11 × 10^9^/l). Blue dashed line—lower limit of normal TLC (4 × 10^9^/l).

In parallel, her total leukocyte count (TLC) was within the normal range at 9.4 × 10^9^/l (normal: 4–11 × 10^9^/l) on presentation, supporting the decision for conservative management. However, it rose to 15.6 × 10^9^/l the following day, then declined steadily throughout her hospital stay. The final recorded TLC was 5.9 × 10^9^/l, 2 days before discharge (Fig. 2).

**Figure 3:**
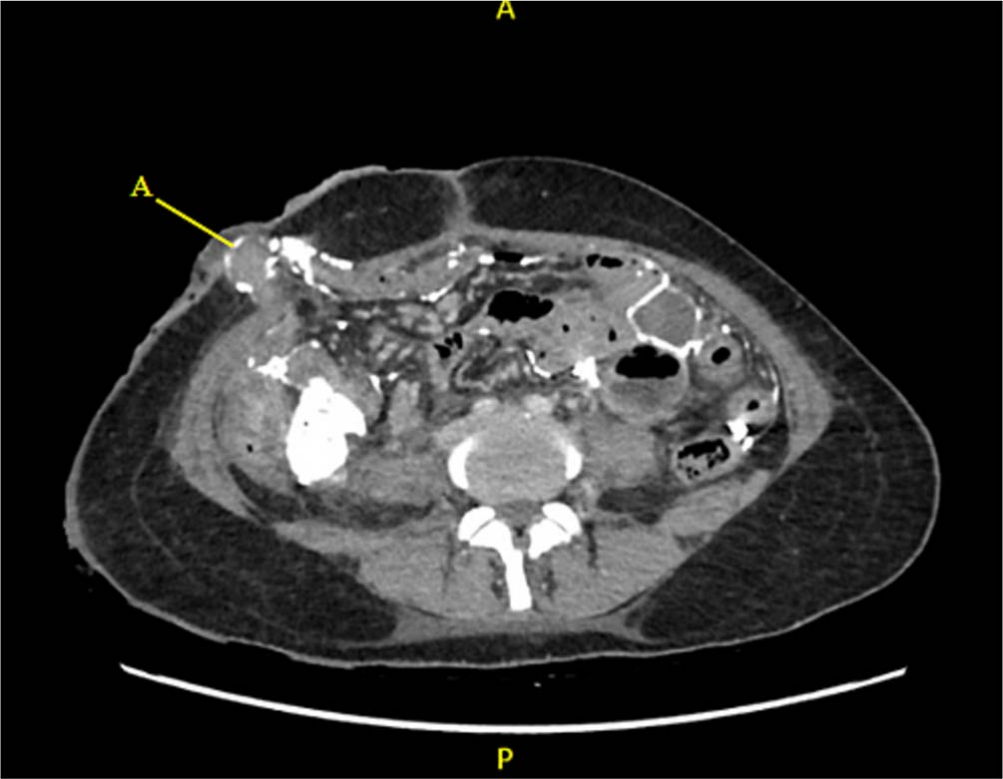
CT contrast abdomen and pelvis showing spill of barium outlining the peritoneal cavity. Stoma at the right side of the abdomen (A).

**Figure 4:**
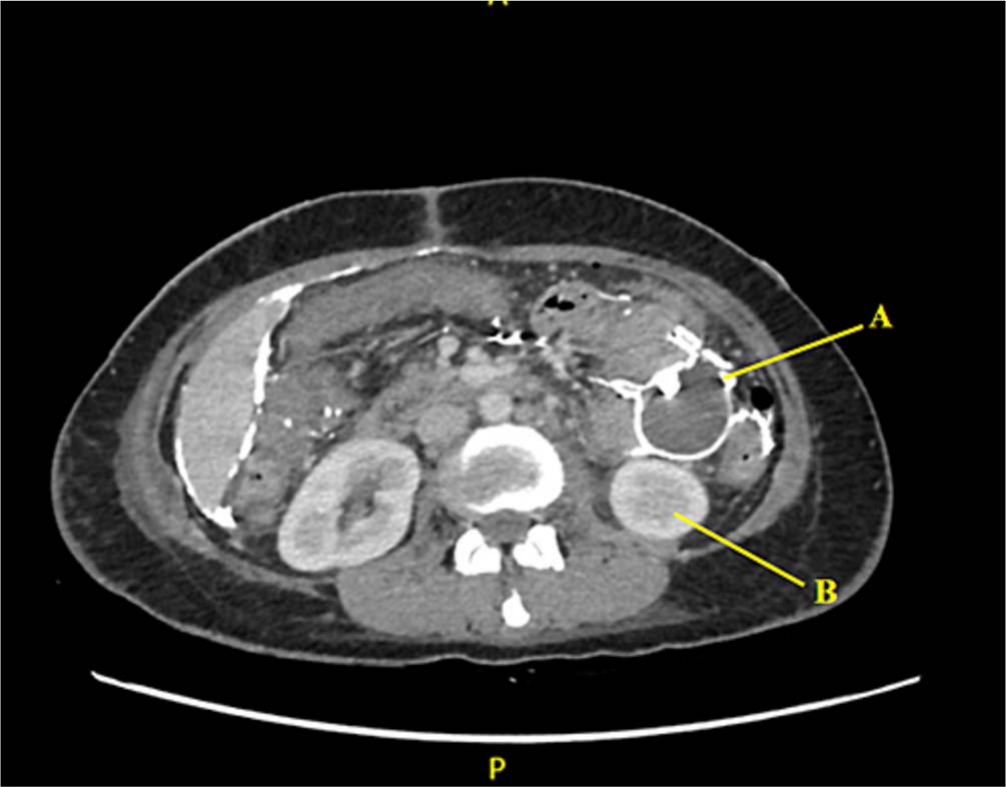
Barium-lined intra-abdominal collection (A) anterior to the left kidney (B).

**Figure 5:**
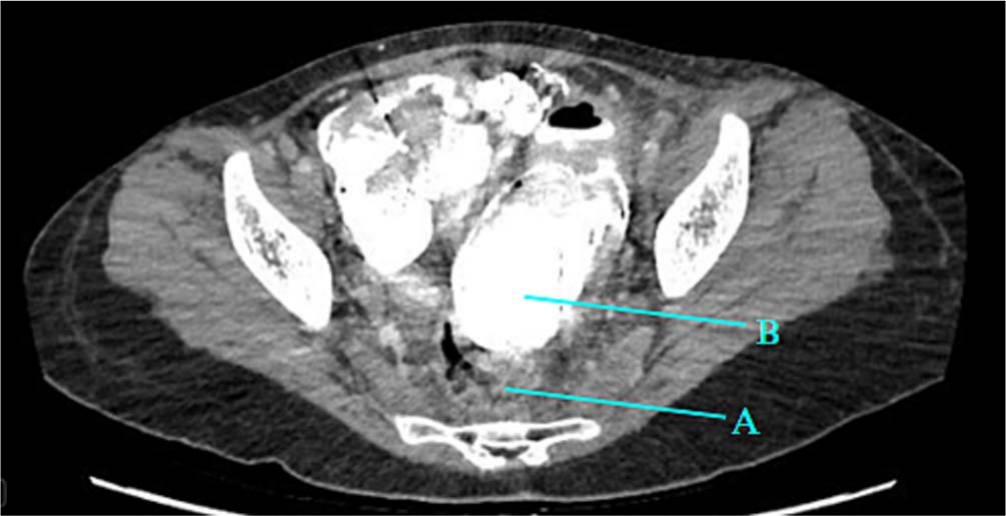
Rectum (A) large barium containing pelvic collection anterior to the rectum, the drain was placed in this collection (B).

Contrast-enhanced CT of the abdomen and pelvis revealed extensive barium extravasation outlining the peritoneal cavity, accompanied by pneumoperitoneum (Fig. 3). Two barium-outlined, walled-off intra-abdominal collections were identified: one located anterior to the lower pole of the left kidney and the other in the pelvis, anterior to the rectum (Figs 4 and 5). Due to significant barium-related imaging artifacts, the precise site of bowel perforation was not identified. A diagnosis of peritonitis secondary to barium spillage was established, with associated intra-abdominal collections.

Given the patient's hemodynamic stability, normal TLC count, and localized abdominal tenderness, a trial of conservative management was initiated. She was managed with intravenous (IV) hydration, analgesia, and broad-spectrum antibiotics. She was initially kept nil per oral, her diet was gradually advanced to regular meals which she tolerated without difficulty. Her ileostomy output was closely monitored. During the initial phase of her hospital stay, her stoma output was high, necessitating the initiation of loperamide, which successfully stabilized the output over time.

**Figure 6:**
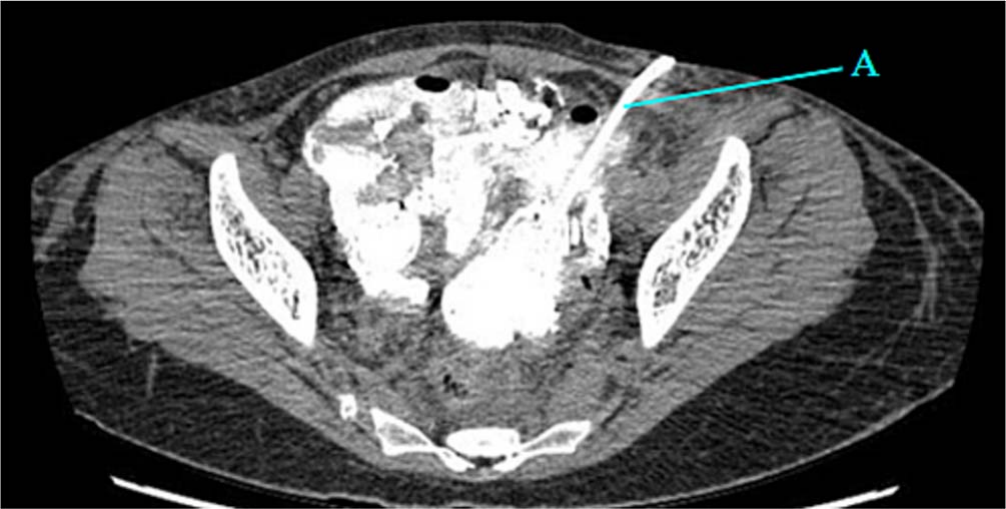
Drain placed in the collection (A).

CT-guided percutaneous drainage was performed for the intra-abdominal collections (Fig. 6), and cultures of the drained fluid revealed heavy growth of *Escherichia coli* and *Enterococcus* species. Her medical history also revealed incomplete antibiotic treatment for the preceding typhoid infection, prompting the initiation of empiric meropenem. Following consultation with the infectious diseases team, her antibiotic regimen was tailored to include tigecycline based on culture sensitivities and her typhoid history.

The patient showed significant clinical improvement, with resolution of fever and abdominal pain. Her CRP count also improved significantly during the hospital stay. She was discharged 9 days after admission with the percutaneous drain in place.

One week after, at her follow-up clinic visit, the patient was asymptomatic with no complaints of abdominal pain or fever. She was advised to focus on nutritional rehabilitation and regular ambulation. Antibiotics were gradually discontinued according to the infectious diseases team’s plan. The drain was subsequently removed once output ceased.

**Figure 7:**
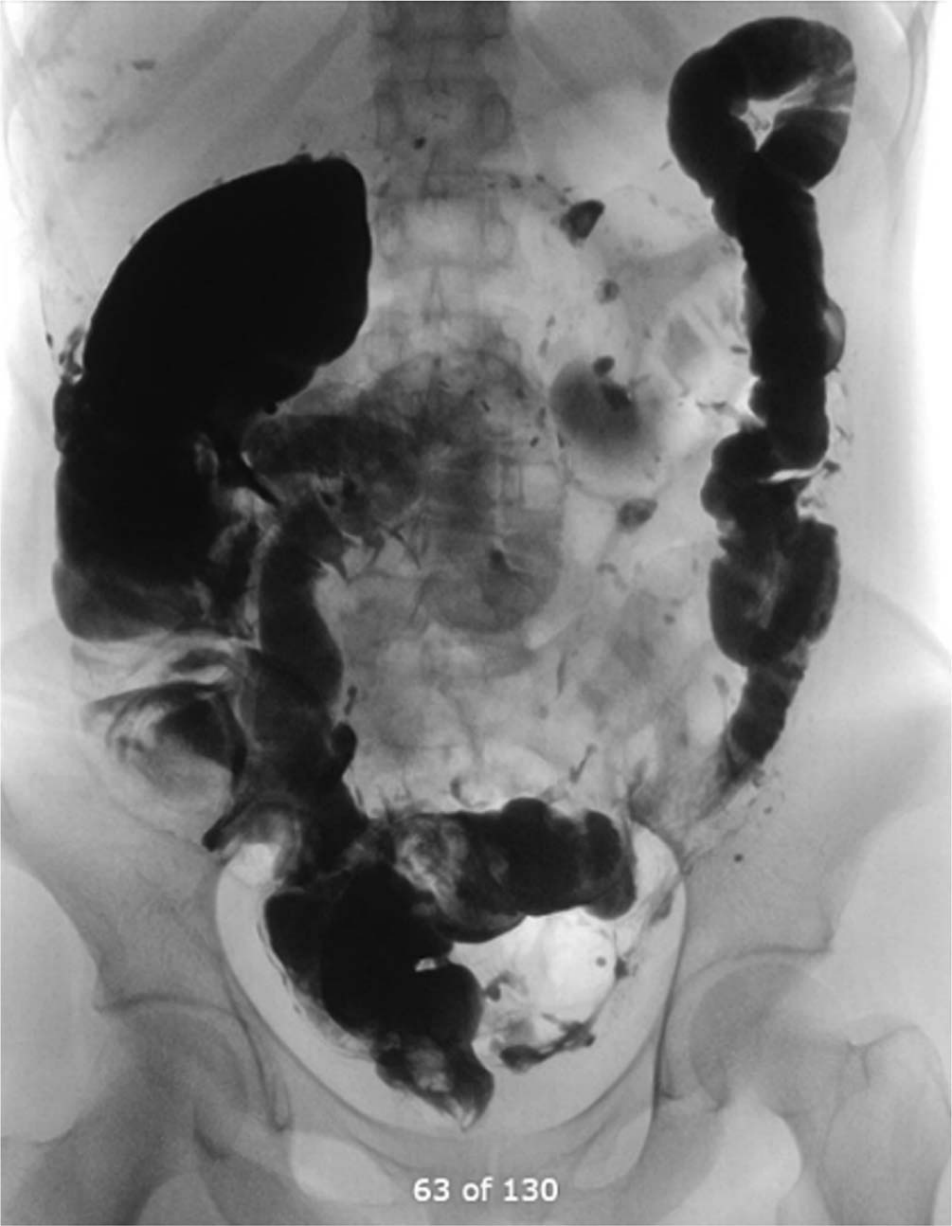
Distal loopogram showing free flow of contrast.

Six weeks later, a distal loopogram was performed using water-soluble gastrogafin contrast, which demonstrated free flow of contrast with no evidence of strictures or leakage (Fig. 7). The patient successfully underwent reversal of ileostomy 2 months afterward and recovered uneventfully.

## Discussion

Barium peritonitis is a rare yet life-threatening complication that can arise following gastrointestinal studies involving barium contrast. While historically managed through surgical intervention, recent advancements in understanding its pathophysiology have allowed for a more nuanced approach to management. In this case, we opted for a conservative approach. This is unlike previous approaches found throughout literature where treatment of this pathology often involved emergent invasive procedures and prolonged hospital stays [1, 2, 5, 6]. Traditionally barium peritonitis has been treated with emergent surgical interventions; however, many cases of acute diverticular perforations with clinically stable patients and localized peritonitis have been successfully managed conservatively. These patients are thought to have sealed off perforations. One systemic review has reported the success rates can be as high as 94% [7]. As our patient had a similar clinical picture, a similar management plan was followed, with a successful outcome.

In our case, the patient remained vitally stable, and her TLC was relatively constant at normal levels throughout her stay, along with the fact that she did not develop generalized peritonitis at any point which may have prompted the decision of urgent surgical intervention. Consequently, the patient recovered uneventfully and was discharged merely 9 days after admission.

Our patient responded well to analgesics and antibiotics, and collection was drained via CT-guided percutaneous drainage. This opens room for future clinical scenarios where patients with localized barium peritonitis probably having a sealed off perforation are spared invasive treatment and managed non-operatively, although a protocol of strict monitoring is required, and laparotomy must be performed in case of failure of non-operative management.

Historically, barium has been used in contrast studies since 1910 [8], however, it has consistently presented with mild to severe adverse effects profile. Gastrointestinal symptoms such as nausea, vomiting, diarrhea, constipation, and cramps have been widely reported [9, 10]. Severe complications involving chemical peritonitis and intestinal obstruction have also been observed. This comes from barium’s ability to clump together with fibrin-rich substances and adhering to the mucosal lining of the bowel thereby precipitating or further exacerbating any previous intestinal obstruction [2].

This raises discussion about whether the application of barium should be continued in modern medicine, especially with the increasing availability of more low-risk water-soluble contrast mediums [11]. Although further clinical trials involving humans are needed. James *et al*. [12] demonstrated that gastrogafin instilled in the peritoneal cavity of domestic cats produced no significant histological reaction or granuloma formation when it came in contact with the peritoneum. Future trials held with human subjects may demonstrate a similar response, thus making gastrogafin a preferred contrast in gastrointestinal radiology. Although gastrogafin may produce a lower quality image because of dilution, its adverse effect profile appears to be entirely tamer than that of barium [11]. Use of barium in patients with suspected perforations and obstruction should be wholly eliminated, as the risk this puts the patient at severely outweighs the benefits, and alternative contrast mediums should be considered.

## Conclusion

This case highlights the successful non-operative management of barium peritonitis in a young female patient, emphasizing the importance of individualized care based on clinical stability, strict monitoring, and multidisciplinary collaboration. Conservative treatment, including IV antibiotics, supportive care, and radiologically guided drainage—can be a viable alternative to surgical intervention in selected cases of barium peritonitis.
